# Effects of postoperative educational interventions on psychological, self-management, and quality-of-life outcomes after coronary artery bypass grafting: a systematic review and meta-analysis

**DOI:** 10.3389/fcvm.2026.1866764

**Published:** 2026-07-15

**Authors:** Shanshan Si, Chaoqian Li, Miaomiao Qi, Jiuxin Ge

**Affiliations:** 1 Department of Nursing, The Fourth Affiliated Hospital of School of Medicine, and International School of Medicine, International Institutes of Medicine, Zhejiang University, Yiwu, China; 2 Department of Cardiology, The Fourth Affiliated Hospital of School of Medicine, and International School of Medicine, International Institutes of Medicine, Zhejiang University, Yiwu, China

**Keywords:** coronary artery bypass grafting, postoperative educational, psychological, quality-of-life, self-management

## Abstract

**Background:**

Postoperative education is an important component of recovery after coronary artery bypass grafting (CABG), but its overall effects on psychological, self-management, and quality-of-life outcomes remain unclear. This systematic review and meta-analysis aimed to evaluate the effects of postoperative educational interventions in patients undergoing CABG.

**Methods:**

PubMed, Web of Science, Embase, Cochrane Library, Scopus, and CINAHL were searched from inception to March 2026. Randomized controlled trials (RCTs) enrolling adult patients undergoing CABG were included if they compared postoperative educational interventions with routine care. Meta-analysis was conducted using Stata/MP 18.0. Pooled effects were calculated as standardized mean differences (SMDs) with 95% confidence intervals (CIs). A random-effects model was used when substantial heterogeneity was present; otherwise, a fixed-effect model was applied. Leave-one-out sensitivity analyses were performed when appropriate.

**Results:**

Fourteen studies involving 1,669 participants were included. Postoperative educational interventions significantly reduced anxiety (SMD = −0.48, 95% CI: −0.87 to −0.08) and depression (SMD = −0.42, 95% CI: −0.71 to −0.14), and significantly improved physical functioning (SMD = 0.30, 95% CI: 0.08 to 0.52). Mental functioning showed a favorable but non-significant trend (SMD = 0.26, 95% CI: −0.01 to 0.53). The pooled effect on self-efficacy was not statistically significant (SMD = 1.08, 95% CI: −1.12 to 3.29), with substantial heterogeneity. Self-power improved significantly (SMD = 0.54, 95% CI: 0.19 to 0.89), but this finding was based on only two studies. Subgroup analyses suggested that intervention effects varied according to the dominant intervention format.

**Conclusions:**

Postoperative educational interventions appear to reduce anxiety and depression and improve physical functioning in patients after CABG. However, the current evidence does not support significant improvements in mental functioning or self-efficacy. Given the limited number of included studies, the finding for self-power should be interpreted cautiously.

## 1 Introduction

Cardiovascular disease remains the leading cause of death worldwide and caused approximately 19.91 million deaths globally in 2021 (1). Coronary heart disease remains a major contributor to this burden, and coronary artery bypass grafting (CABG) continues to play a central role in revascularization for selected patients with complex coronary artery disease. Current North American and European guidelines continue to support CABG in significant left main disease and selected multivessel disease (2, 3). Although surgical techniques and perioperative care have advanced, recovery after CABG remains physiologically demanding and can involve adaptive psychosocial challenges and impaired health-related quality of life (4).

Education is particularly important after CABG because cardiac rehabilitation is not limited to exercise training; it also depends on how well patients understand recovery goals and translate them into daily self-management (5). A recent review of postoperative cardiac rehabilitation after open-heart surgery noted that functional improvement is achieved through rehabilitation exercises, education, and consultations, even though participation in rehabilitation remains suboptimal despite increasing evidence of benefit (6). After discharge, patients recovering from CABG must progressively resume activity, manage wounds, adhere to medications, monitor symptoms, and maintain long-term secondary prevention behaviors. Many patients also experience psychological distress and impaired quality of life during recovery, which may reduce their engagement in rehabilitation and self-care (7). In this context, education is important because it links rehabilitation goals to day-to-day behaviors by helping patients interpret symptoms, regain confidence, adhere to treatment, and adapt to life after surgery. Recent systematic reviews further suggest that education-based and eHealth-supported interventions after CABG or cardiac surgery may improve symptom management, psychological outcomes, and quality of life, although the magnitude of benefit varies across intervention models and outcome domains (8, 9).

However, the current evidence base remains fragmented. Existing reviews have mainly examined single modalities, such as eHealth, cardiac rehabilitation, or perioperative education, rather than evaluating postoperative educational interventions after CABG across psychological, self-management, and quality-of-life outcomes in an integrated way. In addition, primary studies vary substantially in intervention timing, delivery mode, programme intensity, and outcome assessment, which makes the overall effect difficult to interpret and limits implementation in routine postoperative care. Therefore, this systematic review and meta-analysis aimed to evaluate the effects of postoperative educational interventions on psychological outcomes, self-management, and quality-of-life outcomes in patients undergoing CABG.

## 2 Methods

This systematic review and meta-analysis was conducted in accordance with the Preferred Reporting Items for Systematic Reviews and Meta-Analyses (PRISMA) statement (10). The study protocol was registered in the International Prospective Register of Systematic Reviews (PROSPERO; registration No. CRD420261379088).

### 2.1 Search strategy

A comprehensive literature search was performed in the following electronic databases: PubMed, Web of Science, Embase, Cochrane Library, Scopus, and CINAHL, from database inception to March 2026. The search strategy was based on three core concepts: coronary artery bypass grafting, education, and discharge/post-discharge care. The main keywords included “coronary artery bypass grafting” OR “CABG”, “education” OR “teaching” OR “training” OR “counseling”, and “discharge” OR “post-discharge”. Equivalent subject headings and free-text terms were adapted for each database as appropriate. The full search strategies for all databases are provided in the Supplementary File S1.

### 2.2 Eligibility criteria

Studies were considered eligible according to the following criteria: (1) Population: adult patients who underwent coronary artery bypass grafting and were able to receive and participate in educational interventions; (2) Intervention: postoperative educational interventions, including but not limited to structured discharge education, telephone counselling, nurse-led education, web-based or eHealth education, and educational components embedded in rehabilitation programmes; (3) Comparator: usual care, routine discharge instructions, standard nursing care, or other non-educational control conditions; (4) Outcomes: at least one of the following outcomes was reported: psychological outcomes (anxiety, depression), self-management-related outcomes (self-care power, self-efficacy), or quality-of-life outcomes (mental functioning, physical functioning); (5) Study design: randomized controlled trials.

In this review, postoperative educational interventions were operationally defined as structured education, counselling or information-based interventions initiated after CABG during postoperative hospitalization, at discharge, or during post-discharge follow-up. These interventions aimed to support postoperative recovery, symptom management, rehabilitation participation, psychological adaptation, or self-management. Rehabilitation-based interventions were eligible only when structured education was clearly described as a core component; purely exercise-based rehabilitation programmes without a clear educational component were not considered eligible.

To improve interpretability and address clinical heterogeneity, eligible interventions were further classified according to their dominant delivery format into three subgroups: rehabilitation education, information education, and tele-education. Rehabilitation education included education combined with rehabilitation or exercise instruction; information education referred to structured written, audio, video, or discharge information; and tele-education referred to telephone, mobile, or remote follow-up based education or counselling.

Studies were excluded if they: (1) did not involve patients undergoing CABG; (2) did not evaluate a postoperative educational intervention; (3) lacked extractable outcome data; (4) were reviews, conference abstracts, editorials, case reports, or study protocols; or (5) were duplicate publications.

### 2.3 Study selection and data extraction

Two reviewers independently screened the titles, abstracts, and full texts of the retrieved studies according to the predefined eligibility criteria. Disagreements were resolved through discussion or consultation with a third reviewer. The study selection process was presented in a PRISMA flow diagram. Data were independently extracted using a standardized form, including author, year, country, sample size, age, follow-up duration, intervention, control, and outcomes.

### 2.4 Risk of bias assessment

The methodological quality of the included studies was independently assessed by two reviewers. For randomized controlled trials, the Cochrane Risk of Bias tool (RoB 2) was applied. Any disagreement was resolved by discussion or adjudication by a third reviewer.

### 2.5 Publication bias and sensitivity analyses

Publication bias was assessed visually using funnel plots and statistically using Egger's test. Sensitivity analyses were performed using a leave-one-out approach when at least three studies were available for an outcome.

### 2.6 Statistical analysis

Meta-analysis was performed using Stata/MP 18.0. For continuous outcomes, pooled effect sizes were calculated as SMDs, with 95% CIs. Statistical heterogeneity was assessed using Cochran's *Q* test and the I^2^ statistic. An I^2^ value of >50% was considered indicative of substantial heterogeneity, in which case a random-effects model was applied; otherwise, a fixed-effect model was used. A *p*value <0.05 was considered statistically significant.

## 3 Results

### 3.1 Study selection

**Figure 1:**
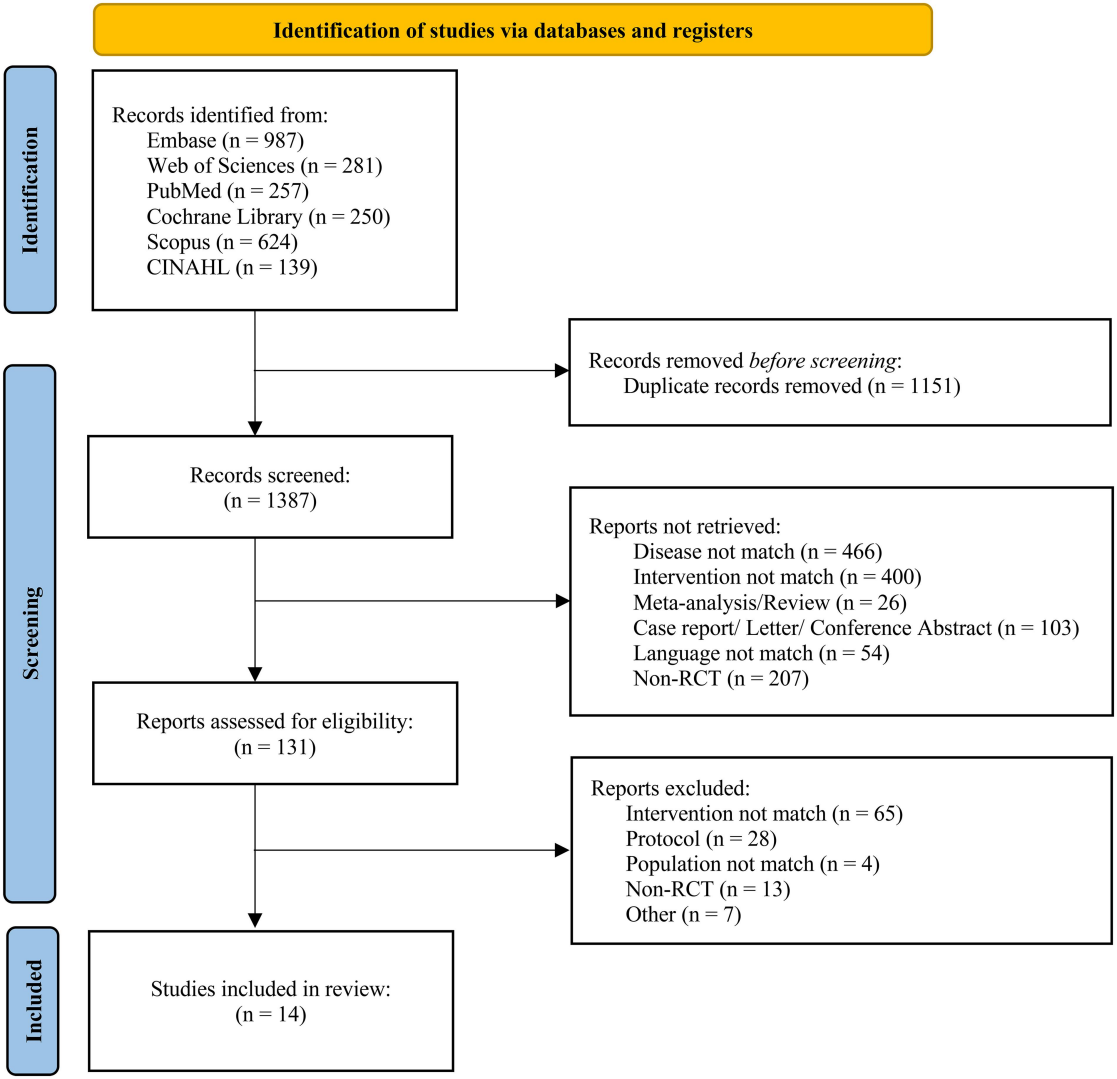
PRISMA flow ndiagram of study selection.

A total of 2,538 records were identified through database searching. After duplicates were removed, 1,387 records remained for screening. Of these, 131 full-text reports were assessed for eligibility, and 14 studies were ultimately included in the systematic review and meta-analysis. The study selection process is presented in Figure 1.

### 3.2 Study characteristics

The main characteristics of the included studies are summarized in Table 1. A total of 14 studies (11–24) involving 1,669 participants were included, with 866 participants in the intervention groups and 803 in the control groups. The studies were published between 1995 and 2024 and were conducted in Australia, Iran, China, the United States, Turkey, Canada, Cyprus, Thailand, France, and Norway. Sample sizes ranged from 60 to 300 participants. Follow-up durations ranged from 3 weeks to 12 months, while one study assessed outcomes only during hospitalization until discharge.

**Table 1:** Characteristics of the included studies.

Author	Year	Country	n		Age		Follow-up duration	Intervention	Control	Outcomes
			I	C	I	C				
Oldenburg B	1995	Australia	43	43	60 ± 7.1	59 ± 8.1	4 months	Behavioral-educational intervention program	Routine Care	①
Sharif F	2012	Iran	40	40	58.4	59.2	2 months	Rehabilitation sessions (educational programs)	Routine Care	①②
Ma L	2020	China	150	150	63.1 ± 9.7	62.8 ± 10.7	12 months	CAD-related health education (coronary artery disease)	Routine Care	①②③④
Mahler HIM	1999	USA	142	72	61.38 ± 8.35		1 month	Videotape information	Routine Care	①
Eskici Ilgin V	2024	Turkey	30	30	58.60 ± 8.75	58.66 ± 10.41	3 weeks	Telephone nursing education and counselling	Routine Care	⑥
Borzou SR	2018	Iran	30	30	61.6 ± 11.7	57.97 ± 13.1	1 month	Cardiac rehabilitation program	Routine Care	⑤
Sawatzky JA	2013	Canada	95	105	64.9 ± 9.3	64.1 ± 9.9	6 weeks	Discharge teaching	Routine Care	③④
Kaya U	2022	Cyprus	35	35	≥50	≥40	2 months	Discharge training and telephone counseling	Routine Care	⑥
Bikmoradi A	2023	Iran	40	40	52.78 ± 6.20	55.75 ± 8.34	6 weeks	Telenursing	Routine Care	①②
Bikmoradi A	2017	Iran	36	35	62 ± 7.41	64.03 ± 7.77	5 weeks	Telephone counseling	Routine Care	③④
Utriyaprasit K	2010	Thailand	60	60	62.78 ± 7.9	63.3 ± 8.17	1 month	Audiotape of information	Routine Care	③④
Moore SM	1999	France	90	90	62 ± 10.8	63.2 ± 10	1 month	Audiotape of information	Routine Care	①②③
Moghaddam NG	2023	Iran	30	30	58.2 ± 8.3	59.1 ± 11.7	Until discharge	Augmented reality-based rehabilitation	Routine Care	⑤
Sørlie T	2007	Norway	45	43	59.0 ± 5.4	57.5 ± 7.2	6 weeks	Video information	Routine Care	①②

① anxiety; ② depression; ③ physical functioning; ④ mental functioning; ⑤ self-efficacy; ⑥ self-power; I, intervention group; C, control group.

### 3.3 Risk of bias

**Figure 2:**
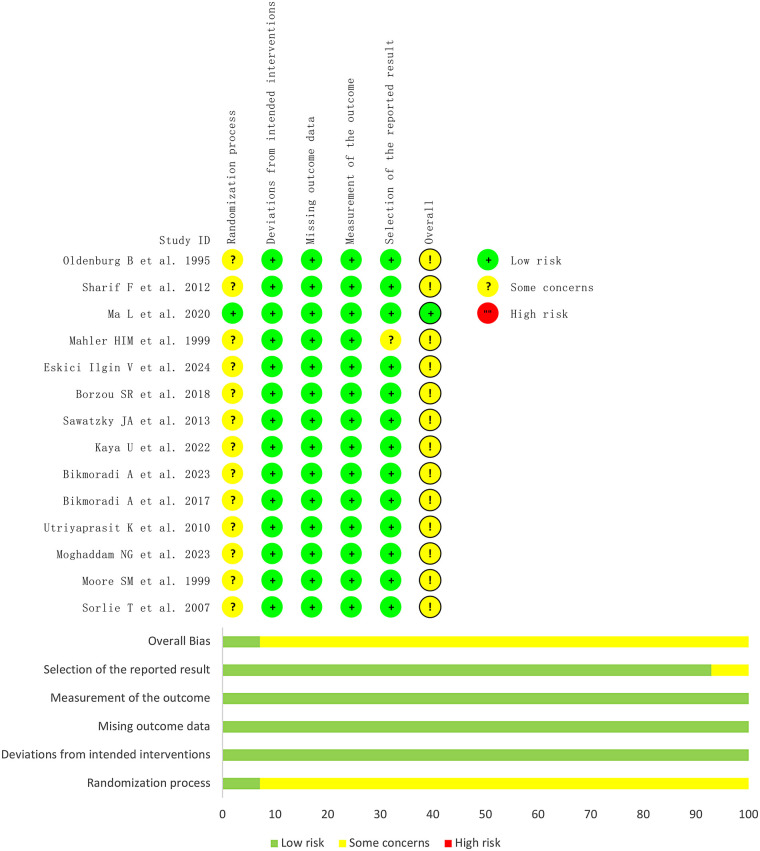
Risk of bias assessment of the included studies.

The methodological quality of the included studies was assessed using the Cochrane Risk of Bias 2 tool. Overall, 1 of the 14 included studies was judged to be at low risk of bias, whereas the remaining 13 studies were rated as having some concerns; no study was judged to be at high risk of bias. Across domains, all studies were rated as low risk for deviations from intended interventions, missing outcome data, and measurement of the outcome. Some concerns arose mainly from the randomization process, which affected 13 studies, and from selection of the reported result in 1 study. The detailed results of the risk of bias assessment are presented in Figure 2.

### 3.4 Results of meta-analysis

#### 3.4.1 Anxiety

**Figure 3:**
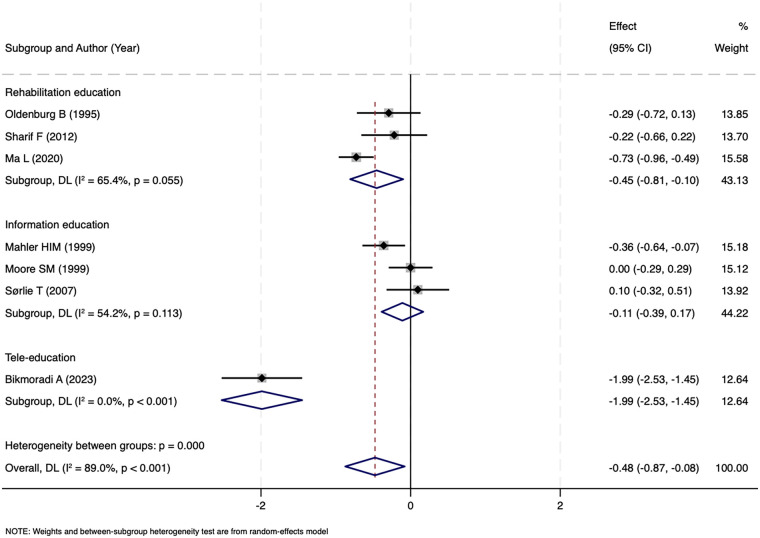
Forest plot of anxiety.

A total of seven studies reported anxiety outcomes. Because substantial heterogeneity was observed (I^2^ = 89.0%, *p* < 0.001), a random-effects model was used. The pooled analysis showed that postoperative educational interventions significantly reduced anxiety compared with the control group (SMD = −0.48, 95% CI: −0.87 to −0.08) (Figure 3). Subgroup analysis showed that rehabilitation education significantly reduced anxiety (SMD = −0.45, 95% CI: −0.81 to −0.10), whereas information education showed no statistically significant effect (SMD = −0.11, 95% CI: −0.39 to 0.17). The tele-education subgroup included only one study and showed a large reduction in anxiety (SMD = −1.99, 95% CI: −2.53 to −1.45). Leave-one-out sensitivity analysis showed that the pooled result remained robust (Supplementary File S2).

#### 3.4.2 Depression

**Figure 4:**
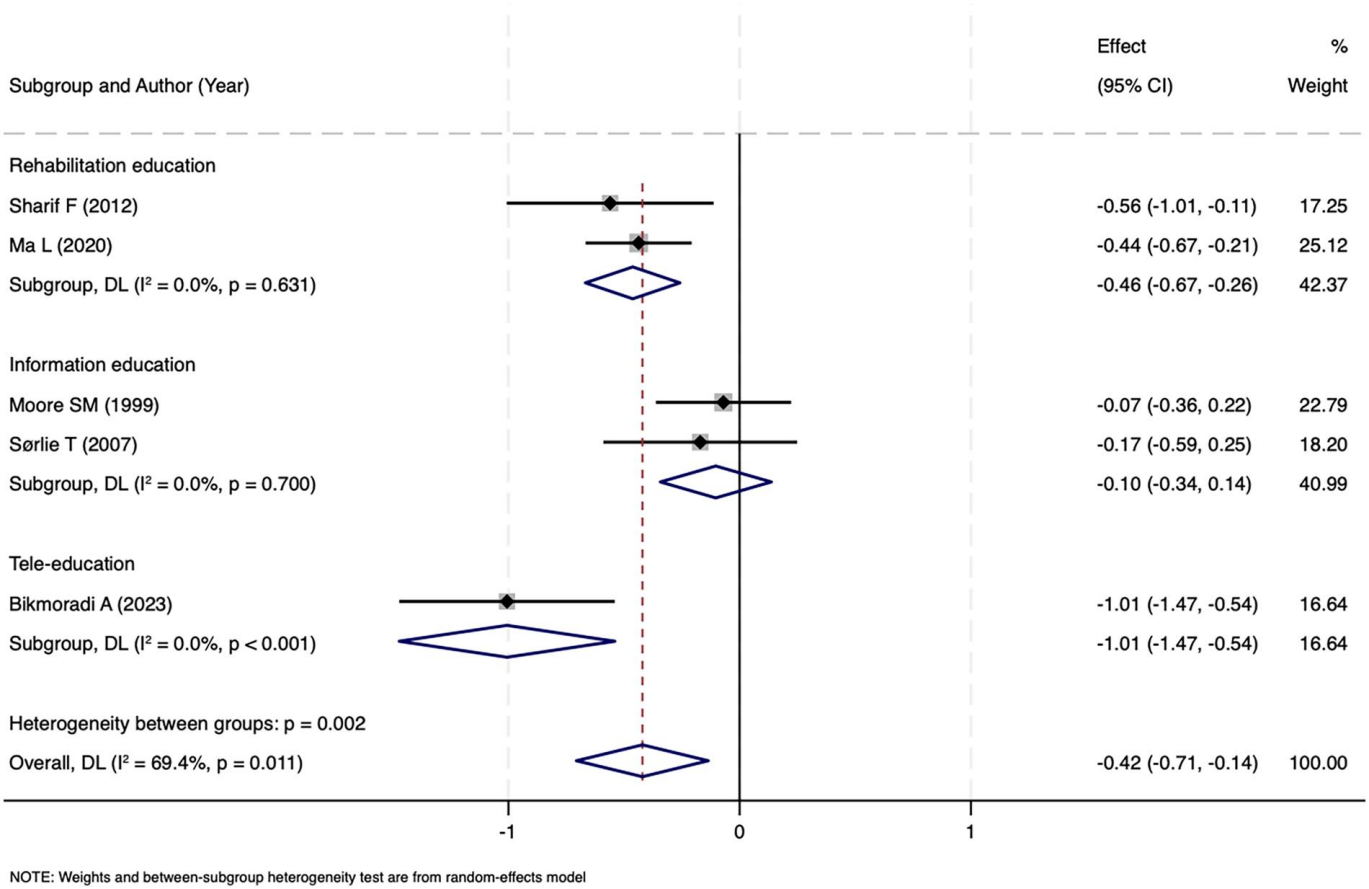
Forest plot of depression.

A total of five studies reported depression outcomes. Because heterogeneity was greater than 50% (I^2^ = 69.4%, *p* = 0.011), a random-effects model was used. The pooled analysis showed that postoperative educational interventions significantly reduced depression compared with the control group (SMD = −0.42, 95% CI: −0.71 to −0.14) (Figure 4). In the subgroup analysis, rehabilitation education was associated with a significant reduction in depression (SMD = −0.46, 95% CI: −0.67 to −0.26), whereas information education showed no statistically significant effect (SMD = −0.10, 95% CI: −0.34 to 0.14). The tele-education subgroup included only one study and showed a significant reduction in depression (SMD = −1.01, 95% CI: −1.47 to −0.54). Leave-one-out sensitivity analysis showed that the pooled result remained robust (Supplementary File S2).

#### 3.4.3 Mental functioning

**Figure 5:**
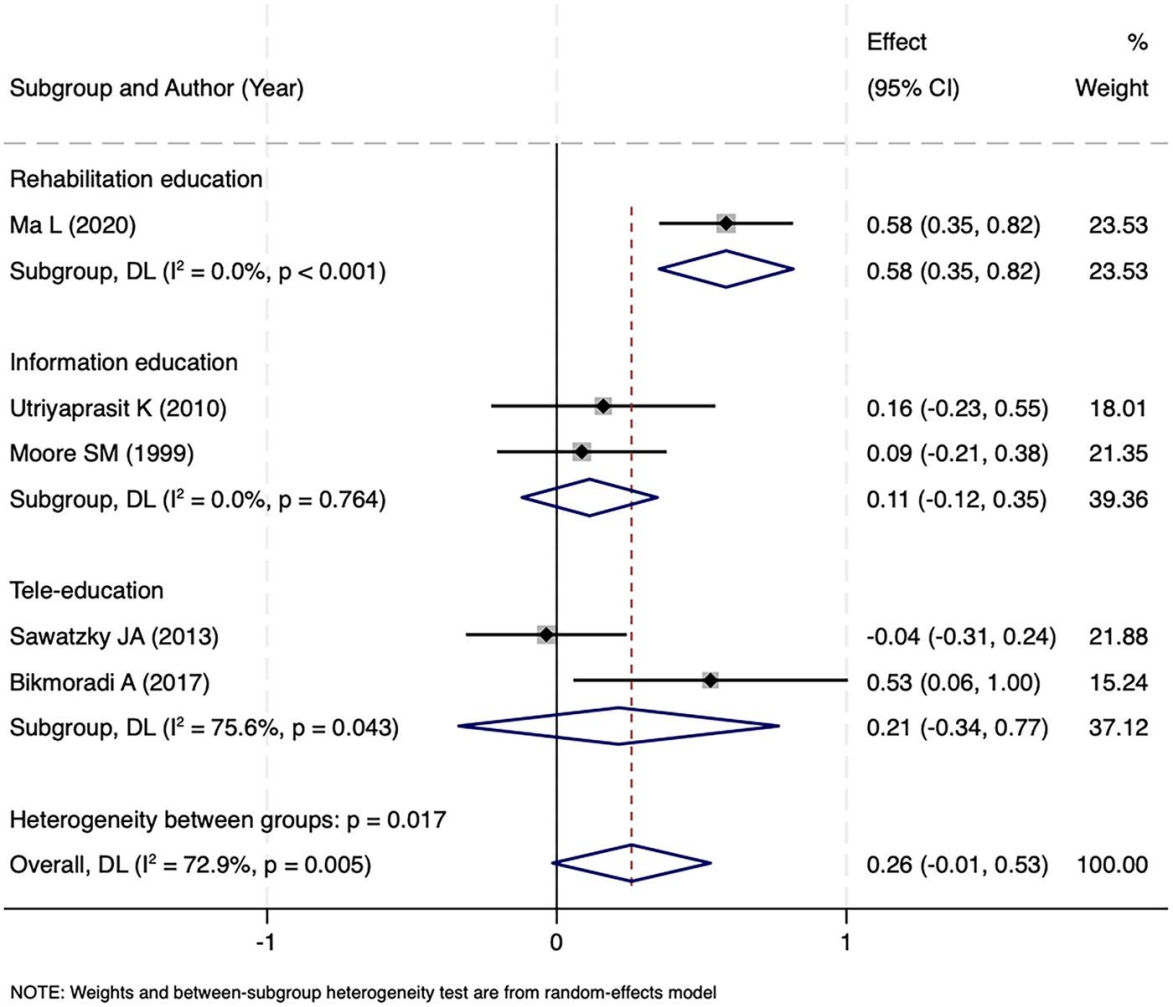
Forest plot of mental functioning.

A total of five studies reported mental functioning outcomes. Because substantial heterogeneity was observed (I^2^ = 72.9%, *p* = 0.005), a random-effects model was used. The pooled analysis showed a favorable but non-significant effect of postoperative educational interventions on mental functioning (SMD = 0.26, 95% CI: −0.01 to 0.53) (Figure 5). Subgroup analysis showed a significant effect in the rehabilitation education subgroup, although this subgroup included only one study (SMD = 0.58, 95% CI: 0.35 to 0.82). No statistically significant effects were observed in the information education subgroup (SMD = 0.11, 95% CI: −0.12 to 0.35) or the tele-education subgroup (SMD = 0.21, 95% CI: −0.34 to 0.77). Leave-one-out sensitivity analysis showed that the overall conclusion remained stable (Supplementary File S2).

#### 3.4.4 Physical functioning

**Figure 6:**
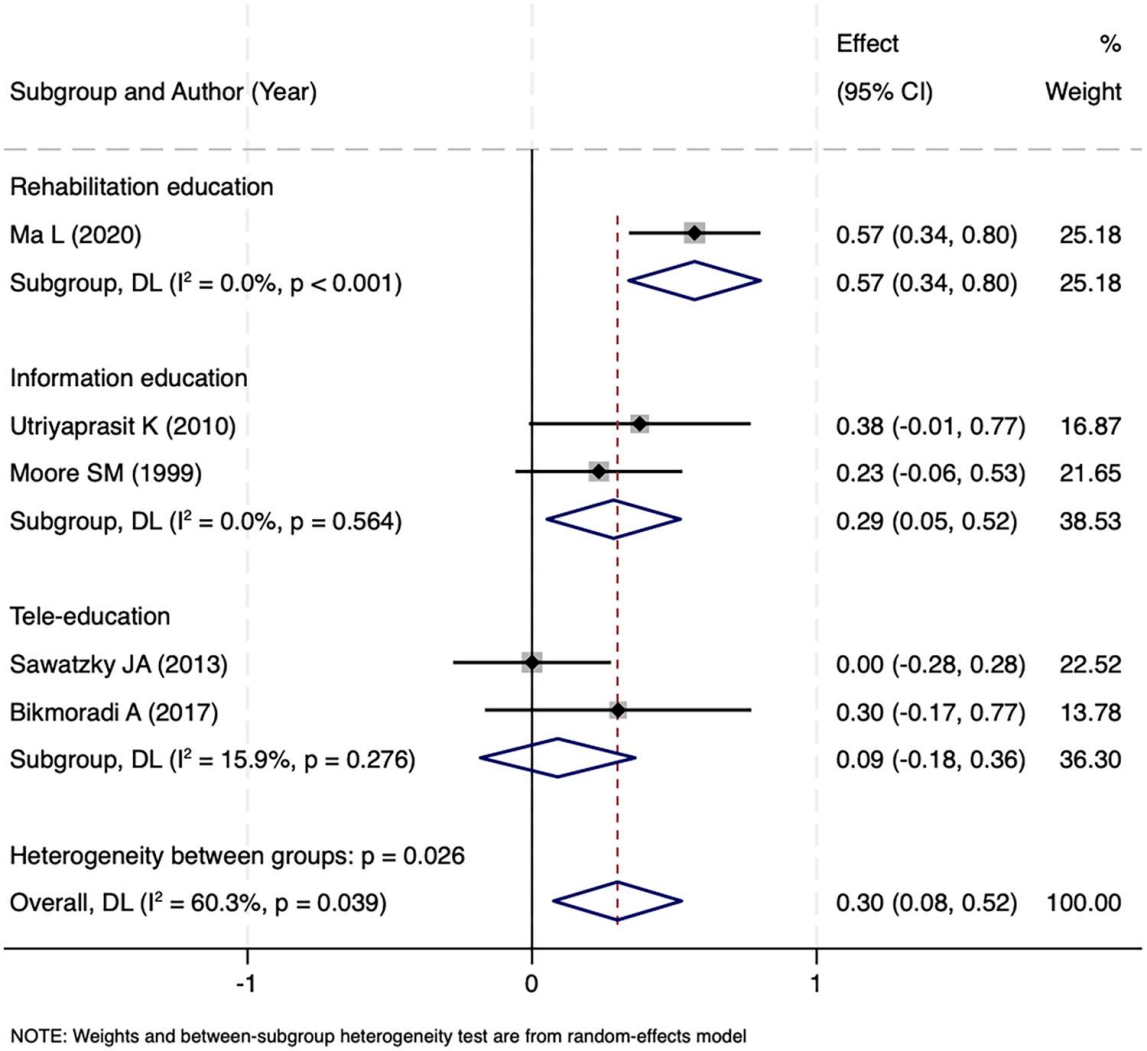
Forest plot of physical functioning.

A total of five studies reported physical functioning outcomes. Because heterogeneity was greater than 50% (I^2^ = 60.3%, *p* = 0.039), a random-effects model was used. The pooled analysis showed that postoperative educational interventions significantly improved physical functioning compared with the control group (SMD = 0.30, 95% CI: 0.08 to 0.52) (Figure 6). In subgroup analysis, rehabilitation education showed a significant improvement in physical functioning, although this subgroup included only one study (SMD = 0.57, 95% CI: 0.34 to 0.80). Information education was also associated with a significant improvement (SMD = 0.29, 95% CI: 0.05 to 0.52), whereas tele-education showed no statistically significant effect (SMD = 0.09, 95% CI: −0.18 to 0.36). Leave-one-out sensitivity analysis showed that the pooled result remained robust (Supplementary File S2).

#### 3.4.5 Self-efficacy

**Figure 7:**
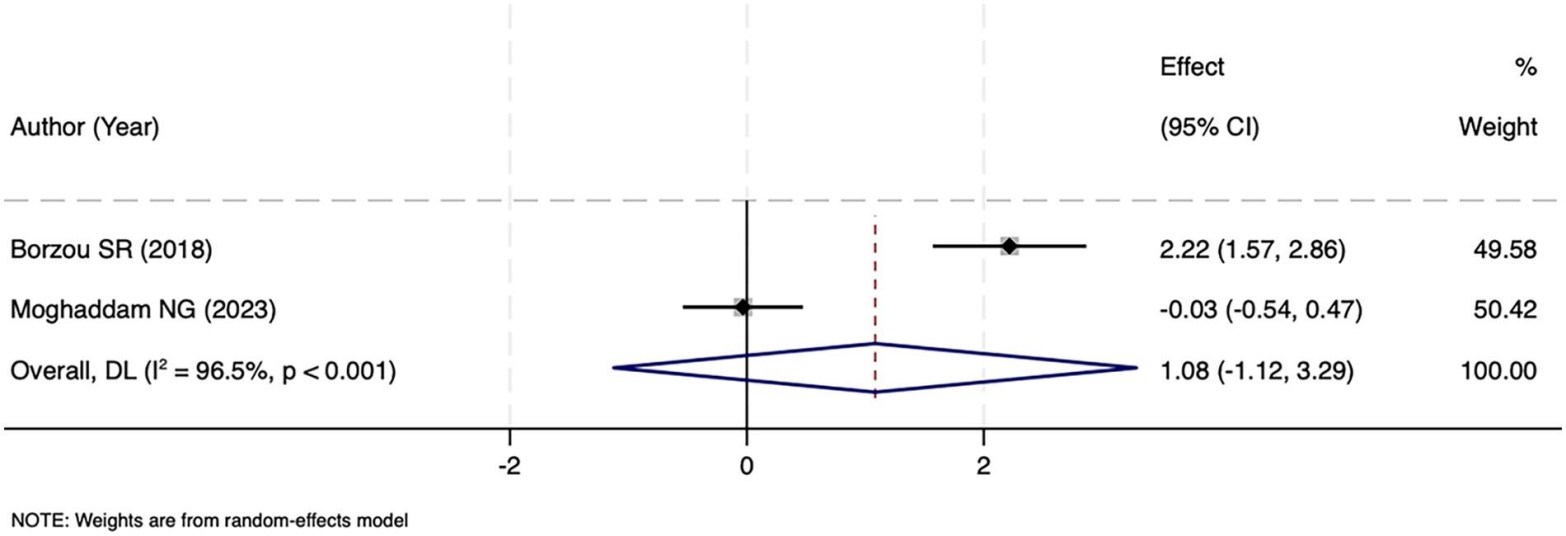
Forest plot of self-efficacy.

A total of two studies reported self-efficacy outcomes. Because substantial heterogeneity was observed (I^2^ = 96.5%, *p* < 0.001), a random-effects model was used. The pooled analysis showed that postoperative educational interventions did not significantly improve self-efficacy compared with the control group (SMD = 1.08, 95% CI: −1.12 to 3.29) (Figure 7). Leave-one-out sensitivity analysis was not considered informative for this outcome because only two studies were included.

#### 3.4.6 Self-power

**Figure 8:**
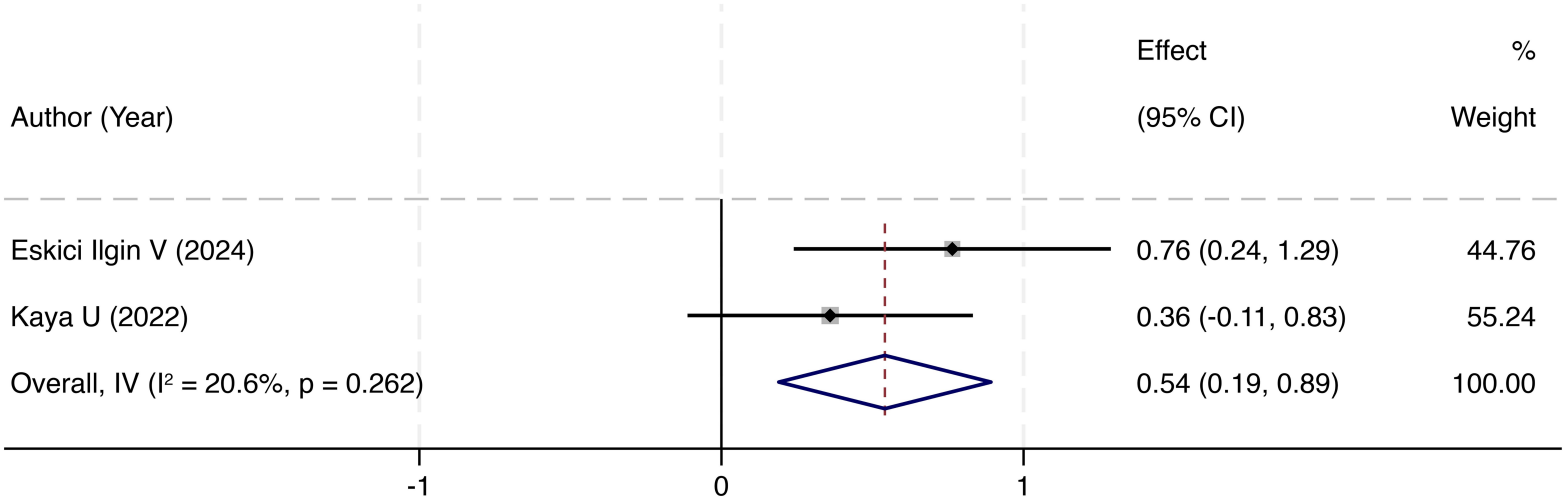
Forest plot of self-power.

A total of two studies reported self-power outcomes. Because heterogeneity was less than 50% (I^2^ = 20.6%), a fixed-effect model was used. The pooled analysis showed that postoperative educational interventions significantly improved self-power compared with the control group (SMD = 0.54, 95% CI: 0.19 to 0.89) (Figure 8). Leave-one-out sensitivity analysis was not considered informative for this outcome because only two studies were included. Therefore, although the pooled effect was statistically significant, the evidence for self-power should be interpreted cautiously because of the limited number of studies.

### 3.5 Publication bias

The funnel plot appeared approximately symmetrical, and Egger’s test did not indicate significant publication bias (*p* = 0.770), suggesting no clear evidence of substantial small-study effects (Supplementary File S3).

## 4 Discussion

This systematic review and meta-analysis evaluated the effects of postoperative educational interventions on psychological, self-management, and quality-of-life outcomes after CABG. Fourteen randomized controlled trials involving 1,669 participants were included. The pooled results showed that postoperative educational interventions significantly reduced anxiety and depression and improved physical functioning. Mental functioning showed a favorable but non-significant trend, whereas the effect on self-efficacy was not statistically significant. Self-power improved significantly, but this finding was based on only two studies and should therefore be interpreted cautiously. Overall, these findings suggest that postoperative education after CABG may be more consistently beneficial for emotional distress and physical recovery than for broader mental functioning and self-management related outcomes.

These findings are generally consistent with previous evidence on cardiac rehabilitation and education-supported interventions after cardiac surgery. Reviews of education-based interventions after cardiac surgery have also reported potential benefits for depressive symptoms, symptom management, and the physical component of quality of life (8, 9). The present review extends this evidence by focusing specifically on postoperative educational interventions after CABG and by synthesizing psychological, self-management, and quality-of-life outcomes within the same review.

The reductions in anxiety and depression are clinically meaningful. Patients recovering from CABG often experience uncertainty about postoperative symptoms, fear of complications, and difficulty adapting to recovery after discharge. Previous evidence has shown that greater depressive symptoms are associated with poorer quality of life after CABG (25). Educational interventions may reduce psychological distress by clarifying the recovery process, helping patients interpret postoperative symptoms, and reducing uncertainty about complications, and activity progression after discharge. In subgroup analyses, rehabilitation education showed significant effects on anxiety and depression, whereas information education did not. This may be because rehabilitation education usually includes not only information provision but also rehabilitation-related instruction and repeated contact with healthcare providers. Tele-education also showed significant effects on anxiety and depression; however, these results were based on a single study in each analysis. Therefore, the subgroup findings should be interpreted as exploratory rather than definitive. Postoperative educational interventions also significantly improved physical functioning. This finding is consistent with the recovery needs of patients after CABG, as patients require guidance on breathing exercises, progressive activity, and safe resumption of daily activities. Education may improve physical functioning by reducing uncertainty about movement and helping patients understand appropriate activity limits during recovery. In subgroup analyses, rehabilitation education and information education were associated with improved physical functioning, whereas tele-education showed no statistically significant effect. This suggests that physical functioning may benefit more from structured rehabilitation guidance and concrete activity-related information than from remote follow-up alone. In contrast, mental functioning did not improve significantly. This does not necessarily mean that educational interventions are ineffective. Rather, broader mental functioning may be less responsive to short-term educational support than more focused outcomes such as anxiety or depression. Mental functioning after CABG is influenced by multiple psychosocial factors beyond education alone, including social support, perceived control, and functional status. Therefore, short-term or moderate-intensity educational interventions may reduce distress without producing a clear improvement in broader mental functioning within a limited follow-up period. This interpretation is consistent with a meta-analysis showing that eHealth interventions after cardiac surgery improved depression and the physical component of quality of life, but not the mental component (8). The evidence for self-management related outcomes remains uncertain. The pooled effect on self-efficacy was not statistically significant, and substantial heterogeneity was observed. Although self-power improved significantly, only two studies contributed data to this outcome. Therefore, the current evidence is insufficient to determine whether postoperative educational interventions consistently improve self-management related outcomes after CABG. Differences in intervention content, outcome measurement, and follow-up duration may partly explain the inconsistent findings. Future studies should use clearer intervention descriptions and more consistent outcome measures when evaluating self-efficacy, self-power, and other self-management related outcomes.

Substantial heterogeneity was observed for several outcomes, and several limitations should be considered when interpreting the findings. The subgroup analyses suggested that intervention format may be an important source of heterogeneity, as rehabilitation education, information education, and tele-education differed in delivery mode, intensity, duration, provider contact, and degree of behavioral practice. Differences in outcome instruments and follow-up duration may also have contributed to variation in effect sizes and may limit the clinical interpretability of pooled SMDs. In addition, only a small number of studies contributed data to some outcomes, especially self-efficacy and self-power, limiting confidence in these findings. The risk of bias assessment should also be considered when interpreting the pooled estimates. Although no study was judged to be at high risk of bias, 13 of the 14 included studies were rated as having some concerns, mainly because of insufficient reporting of sequence generation and allocation concealment. These reporting limitations make it difficult to fully judge whether group assignment was adequately randomized and concealed and therefore may introduce potential selection bias. Such bias could affect the magnitude of pooled effects by exaggerating or attenuating the estimated benefits of postoperative educational interventions. In addition, one study had some concerns regarding selection of the reported result, which may increase the possibility that favorable or statistically significant outcomes were preferentially reported. Despite these limitations, the findings for anxiety, depression, and physical functioning remained stable in sensitivity analyses, supporting the robustness of the main results.

These findings have practical implications for nurses involved in postoperative care after CABG. Nurses should provide structured, recovery-oriented education and integrate it into routine postoperative care rather than limiting education to brief discharge instructions. Educational content should be delivered in a clear and consistent manner, with attention to both emotional reassurance and practical guidance for safe recovery after discharge. For emotional outcomes, nurses may help reduce anxiety and depression by improving patients’ understanding of recovery and reducing uncertainty. For physical functioning, they should provide concrete guidance on rehabilitation-related activities and safe recovery. Nurse-led telephone or remote follow-up may be useful for continuing support after discharge.

In conclusion, postoperative educational interventions may reduce anxiety and depression and improve physical functioning in patients after CABG. However, current evidence does not confirm significant improvements in mental functioning or self-efficacy. Although self-power showed a statistically significant improvement, this finding should be interpreted cautiously because only two studies contributed data. Future randomized controlled trials should provide clearer descriptions of educational interventions, use standardized outcome measures, report randomization procedures more completely, and include longer follow-up to identify which educational strategies are most effective for postoperative recovery after CABG.

## Data Availability

The original contributions presented in the study are included in the article/Supplementary Material, further inquiries can be directed to the corresponding author.

## Supplementary material

Supplementary File S1 Search strategy.

Supplementary File S2 Results of sensitivity analysis.

Supplementary File S3 Results of publication bias.
