# Supplementary material for: Effects of postoperative educational interventions on psychological, self-management, and quality-of-life outcomes after coronary artery bypass grafting: a systematic review and meta-analysis
Source: Front Cardiovasc Med. 2026 Jul 15;13:1866764. doi: 10.3389/fcvm.2026.1866764 (PMC13415878; doi:10.3389/fcvm.2026.1866764)

**Supplementary Materials**

**Search Strategy**

| No | Query |
| --- | --- |
| 1 | (((((((((((((((education[Title/Abstract]) OR (teaching[Title/Abstract])) OR (training[Title/Abstract])) OR (counseling[Title/Abstract])) OR (counselling[Title/Abstract])) OR (telephone nursing education[Title/Abstract])) OR (nurse-led education[Title/Abstract])) OR (nursing education[Title/Abstract])) OR (patient education[Title/Abstract])) OR (educational program*[Title/Abstract])) OR (training program*[Title/Abstract])) OR (self care education[Title/Abstract])) OR (self management education[Title/Abstract])) OR (self care program*[Title/Abstract])) OR (self management program*[Title/Abstract])) OR (individualized self care program[Title/Abstract]) |
| 2 | ((((discharge[Title/Abstract]) OR (hospital discharge[Title/Abstract])) OR (patient discharge[Title/Abstract])) OR (post-discharge[Title/Abstract])) OR (after discharge[Title/Abstract]) |
| 3 | #1 AND #2 |
| 4 | "Coronary Artery Bypass"[Mesh] |
| 5 | (((((((Artery Bypass, Coronary[Title/Abstract]) OR (Coronary Artery Bypass Grafting[Title/Abstract])) OR (Coronary Artery Bypass Graft[Title/Abstract])) OR (Coronary Artery Bypass Surgery[Title/Abstract])) OR (Aortocoronary Bypass[Title/Abstract])) OR (Bypass Surgery, Coronary Artery[Title/Abstract])) OR (Bypass, Coronary Artery[Title/Abstract])) OR (CABG[Title/Abstract]) |
| 6 | #4 OR #5 |
| 7 | #3 AND #6 |

Results of sensitivity analysis


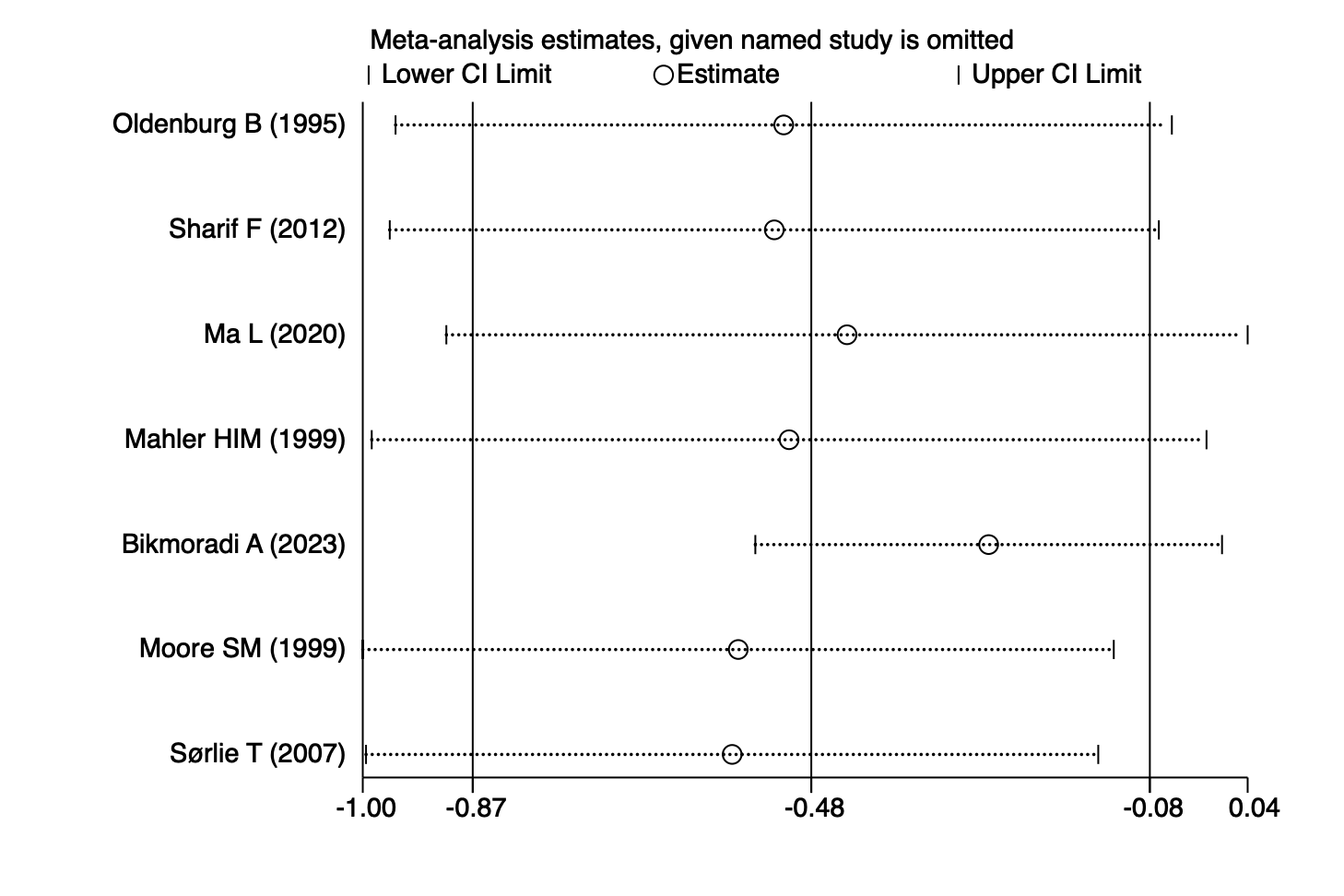


Leave-one-out sensitivity analysis for anxiety


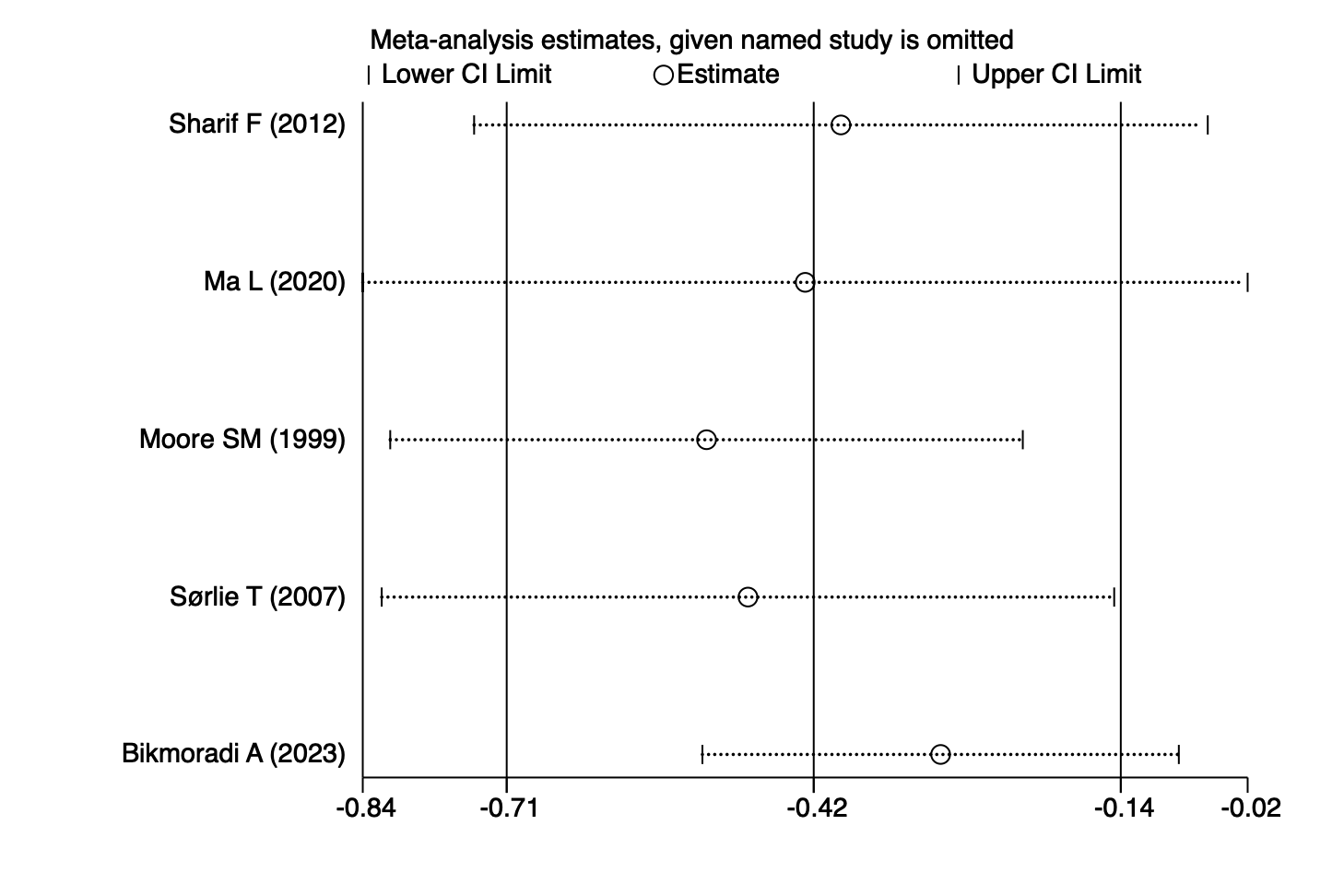


Leave-one-out sensitivity analysis for depression


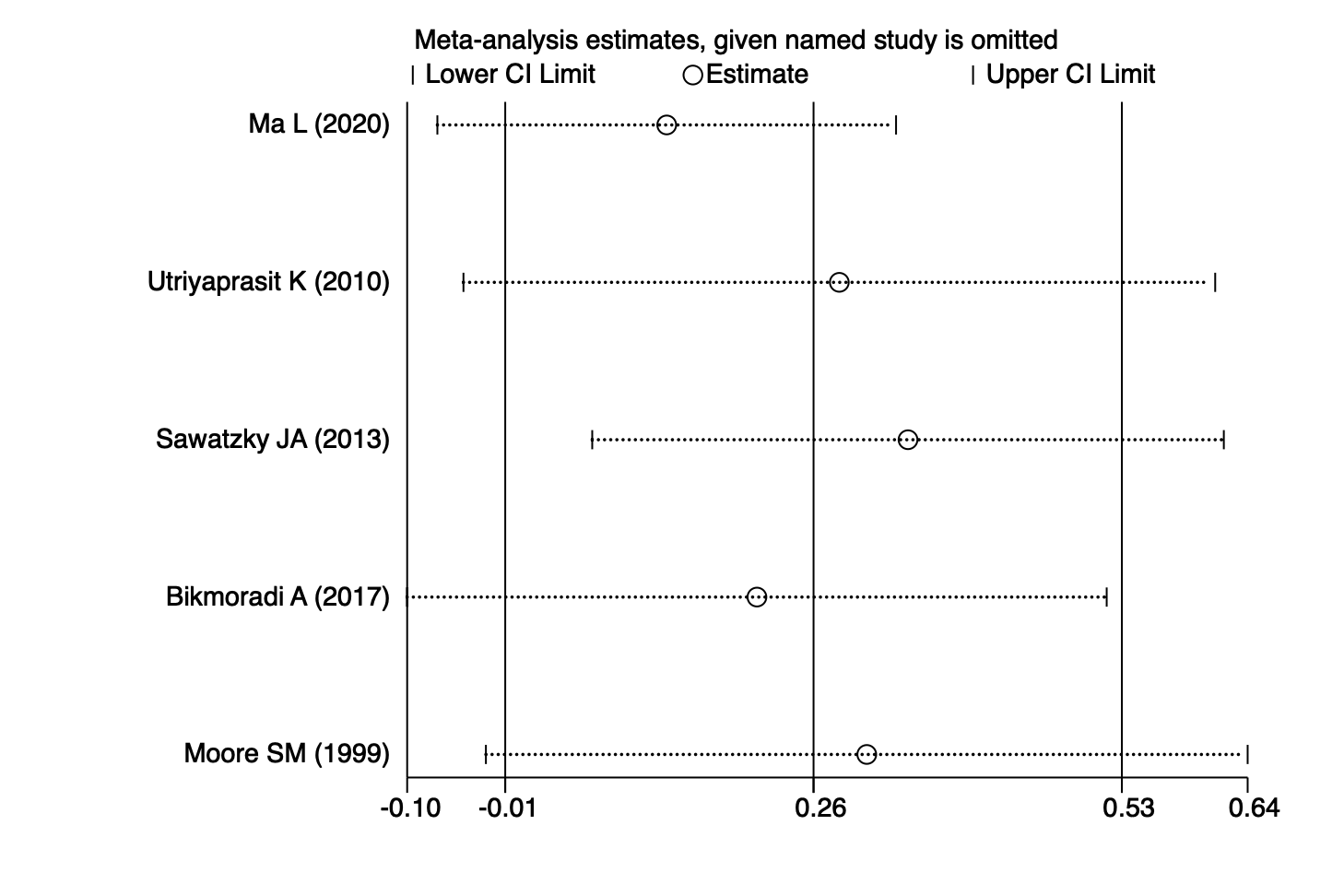


Leave-one-out sensitivity analysis for mental functioning


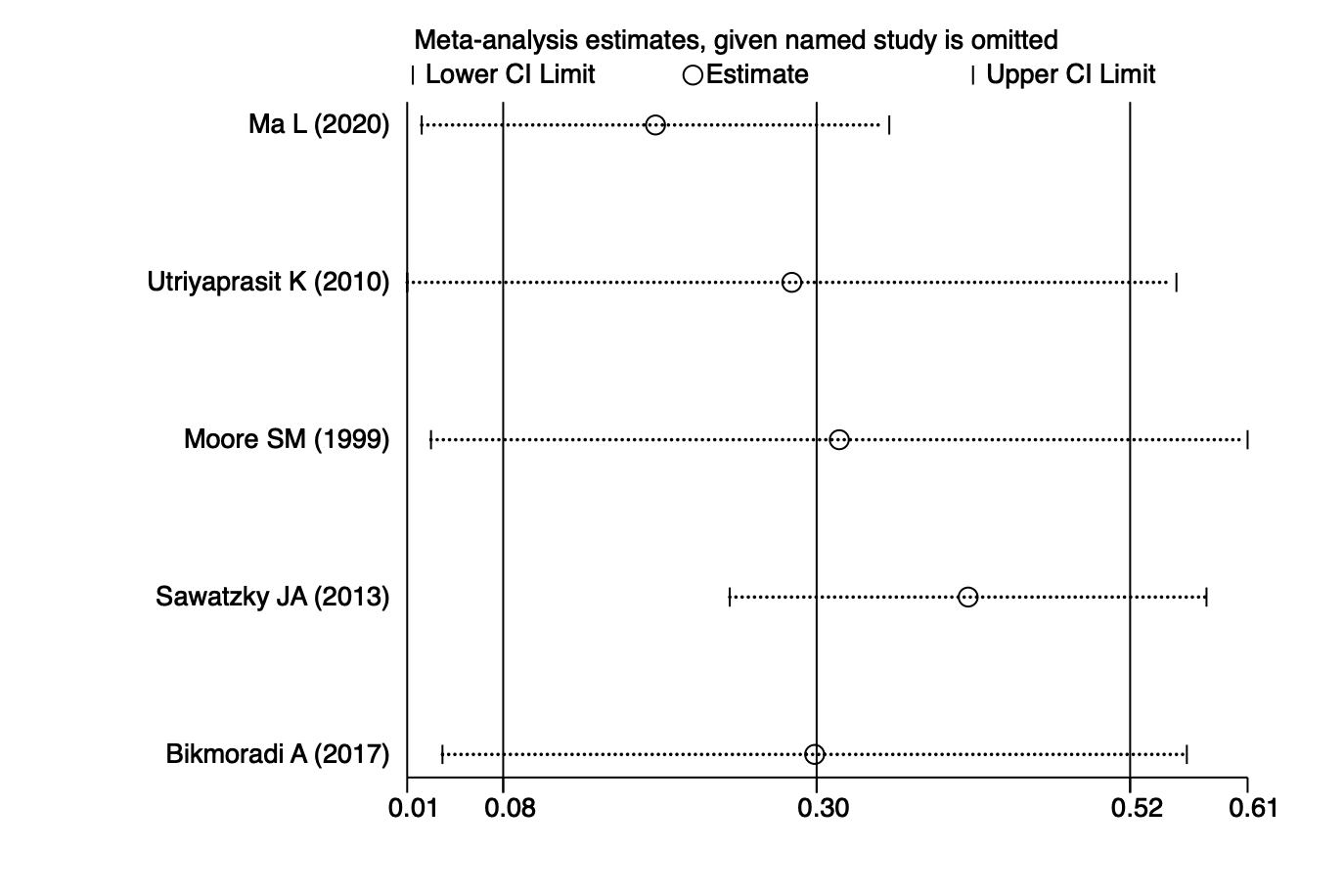


Leave-one-out sensitivity analysis for physical functioning

**Results of publication bias**


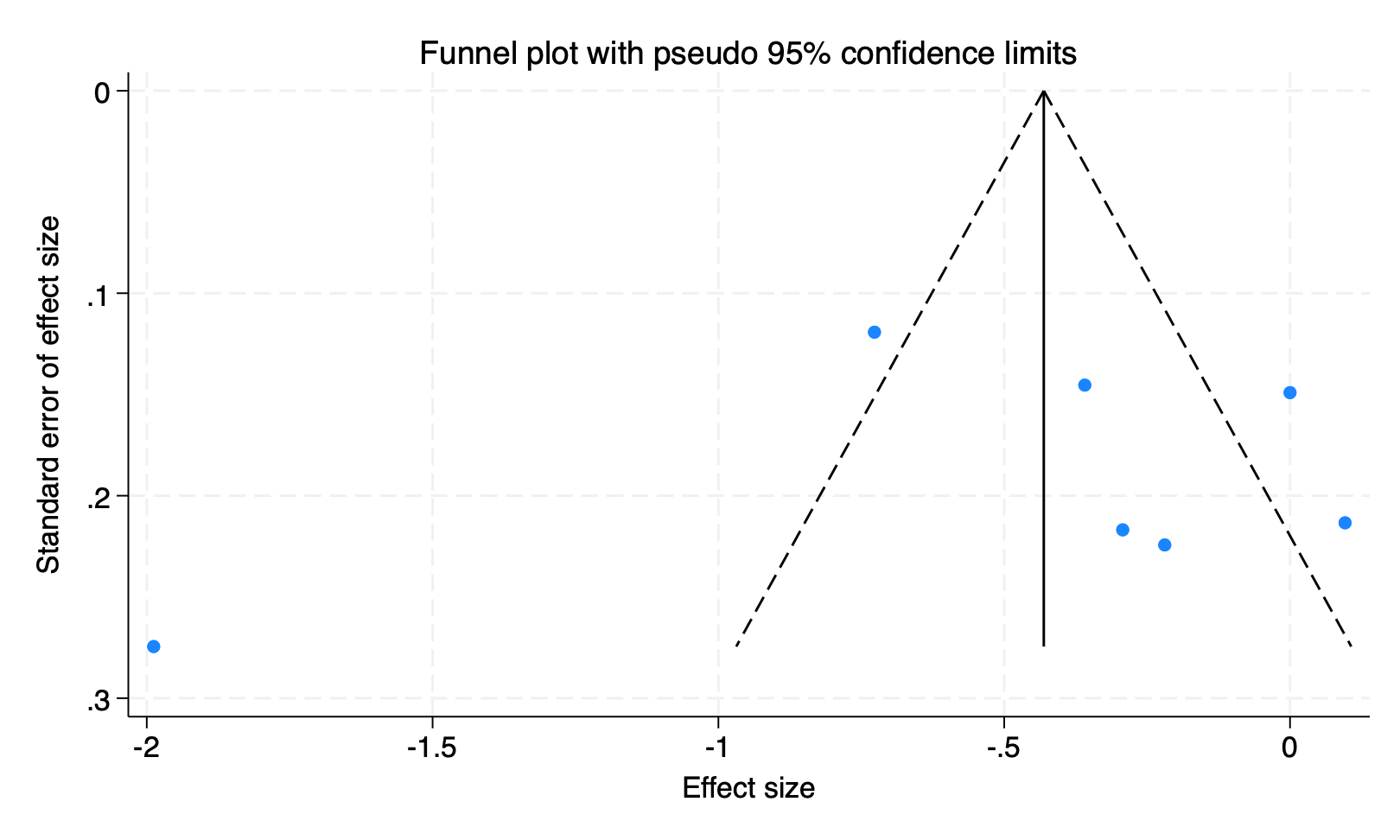


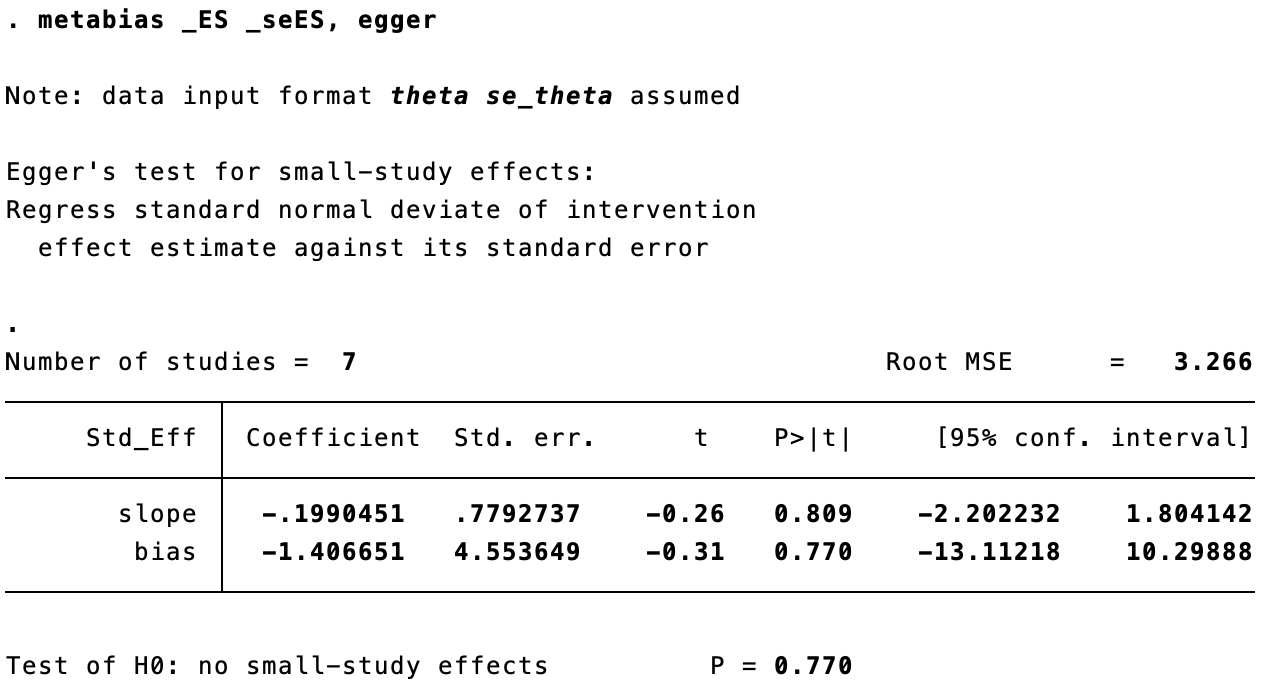

Supplement: Supplementary File S1 — Search strategy. [file Datasheet1.docx]
